# *FLO*5 gene controls flocculation phenotype and adhesive properties in a *Saccharomyces cerevisiae* sparkling wine strain

**DOI:** 10.1038/s41598-017-09990-9

**Published:** 2017-09-07

**Authors:** Paola Di Gianvito, Catherine Tesnière, Giovanna Suzzi, Bruno Blondin, Rosanna Tofalo

**Affiliations:** 1 Faculty of BioScience and Technology for Food, Agriculture and Environment, University of Teramo, Via R. Balzarini 1, 64100 Teramo, Italy; 2 INRA, UMR1083 Science pour l’Œnologie, Montpellier, France; 3 Montpellier SupAgro, UMR1083 Science Pour l’Œnologie, Montpellier, France; 4 Université Montpellier 1, UMR1083 Science pour l’Œnologie, Montpellier, France

## Abstract

Flocculation is an important feature for yeast survival in adverse conditions. The natural diversity of flocculating genes in *Saccharomyces cerevisiae* can also be exploited in several biotechnological applications. Flocculation is mainly regulated by the expression of genes belonging to the *FLO* family. These genes have a similar function, but their specific contribution to flocculation ability is still unclear. In this study, the distribution of *FLO*1, *FLO*5 and *FLO*8 genes in four *S. cerevisiae* wine strains was investigated. Subsequently, both *FLO*1 and *FLO*5 genes were separately deleted in a flocculent *S. cerevisiae* wine strain. After gene disruption, flocculation ability and agar adhesion were evaluated. *FLO*1 and *FLO*5 genes inheritance was also monitored. All strains presented different lengths for *FLO*1 and *FLO*5 genes. Results confirm that in *S. cerevisiae* strain F6789, the *FLO*5 gene drives flocculation and influences adhesive properties. Flocculation ability monitoring after a cross with a non-flocculent strain revealed that *FLO*5 is the gene responsible for flocculation development.

## Introduction

The ability to adhere to other cells and substrates is a crucial feature of microorganisms that can be used both for ‘defensive’ or ‘offensive’ purposes. In *Saccharomyces cerevisiae*, it has the univocal purpose of allowing yeast survival in different biological contexts^1^. Yeast strains display different adhesion phenotypes such as biofilm, invasive growth, agglutination, chain formation co-flocculation and flocculation. Some wine strains have a specific adhesion phenotype called flocculation. This is an asexual, reversible, calcium-dependent process of yeast cells aggregation into flocs that rapidly sediment to the bottom of the liquid growth substrate^2^. The propensity to flocculate is a very significant property of wine yeasts, especially for sparkling wine production with traditional methods. Flocculation offers a more cost-effective method for biomass recovery than centrifugation and filtration, which not only requires high capital investment but also consumes more energy^3^. Indeed, cells that adhere to each other can be conveniently separated from the fermentation product, thus reducing riddling time to only 2 days using automated riddling machines^4^.

During secondary fermentation, wine yeast cells are usually exposed to different stress conditions such as low pH and temperature, high sulphur dioxide level, high total acidity, lack of nutrients and high ethanol content^5^. Flocculation is recognized as a way for cells in solution to escape harsh conditions through sedimentation. Compounds produced during wine fermentation such as ethanol, or the aromatic alcohols tryptophol and phenylethanol, are used in *S. cerevisiae* as quorum sensing molecules^6^. Based on cell density, these compounds act by modulating *FLO* genes regulation^7^. Therefore, flocculation is a cooperative mechanism for protection against multiple chemical stresses^6^. It is mainly due to a variation of cell wall content in fatty acids, ergosterol and trehalose^6, 8^. Furthermore, Goossens *et al*.^9^ found that flocculation contributes to creating specific micro-environmental conditions that favour mating. As mating is a source of genetic variation, diploid cells can thus sporulate and increase the strain’s survival rate.

Flocculation is a two-stage cell-cell adhesion process based equally on glycan-glycan interaction as well as glycan-lectin binding^9^. Initially, there is a glycan-glycan interaction (calcium-binding hypothesis of Mill^10^) reinforced afterwards by specific proteins termed ‘flocculins’ or ‘adhesins’^3^, ‘lectins’^2, 11^ or ‘zymolectins’^12^ (lectin-like theory of Miki *et al*.^11^). These proteins protrude from the cell surface and directly bind to mannose residues present on the cell wall of neighbour cells. In any case, the presence of Ca^2+^ ions is very important because these ions are directly involved in the binding with sugars^9^ and they allow maintaining the correct conformation of flocculins^11^. Recently, Goossens *et al*.^9^ demonstrated that flocculins are drivers of a homophilic interaction, which ensures species-specific aggregation^6^. Floc structure is preferentially composed of *FLO*-expressing cells, probably because of reciprocal interactions. Flo proteins are exclusively responsible for flocculation because they are the most abundant cell wall proteins^13^, so that binding with glycans from others cell wall proteins is not easily achieved. Furthermore, Flo proteins can only interact with mannans present on adjacent cells: indeed, these adhesins are characterised by the presence of two binding sites that immobilise the N-terminal extremity at the cell surface, and by a low binding affinity ensuring that any occasional binding between flo proteins in the same cell is prevented^9^.

Flocculins share a common structure that consists of three domains: i) the carboxyl-terminal domain containing a glycosylphosphatidylinositol anchor that allows flocculin binding to the cell surface^14, 15^ ii) the central domain, rich in serine and threonine residues and heavily glycosylated, is characterized by a large number of tandem repeats that trigger frequent slippage and/or recombination events during DNA replication^16^ iii) the N-terminal effector region, termed CRD (Carbohydrate-Recognition Domain)^17^.

Members of the Flo adhesin protein family in *S. cerevisiae* can be subdivided into two groups^18^. The *FLO*1, *FLO*5, *FLO*9, and *FLO*10 genes encode the members of the first group of proteins. These adhesins promote cell-cell adhesion and contribute to the formation of multicellular clumps (flocs). The second group includes Fig2p and Aga1p that are induced during mating^19, 20^, as well as Flo11p (also known as Muc1p) that is required for diploid pseudohyphal formation and haploid invasive growth^21, 22^. Besides, *FLO*8 is a transcriptional activator essential for *STA*1^23^, *FLO*11^24^, *FLO*1^25^ and *FLO*9^3^ inducible expression. Most laboratory strains, such as S288C and W303-1A, lack the *FLO*8 gene, so they hardly flocculate^26^.

Several studies have nevertheless revealed that there is a high biodiversity of flocculation phenotypes and genotypes among industrial^27, 28^, wine^29^, cachaça^30^ and brewery^31^ strains of *S. cerevisiae*. Correspondingly, Verstrepen *et al*.^32^ highlighted the role of the number of tandem repeats in the central domain of flocculins in the diversity of phenotypes.

The genes of the first group (*FLO*1, *FLO*5, *FLO*9 and *FLO*10) share considerable sequence homology, but the evolutionary advantage linked to the existence of a large family of genes involved in the same phenotype is still unclear^33^. It could be partly explained by the fact that different *FLO* genes confer quite different flocculation degrees^16, 34^, or encode proteins that have different responses to proteases or heat treatments^35^ or still, bind to different sugars^9, 34, 36^. Furthermore, the subtelomeric position of *FLO* genes makes them more liable to undergo recombination events^32^.

In a previous work, flocculation ability has been reported as a not uncommon feature in *S. cerevisiae* wine strains (i.e. in more than 6.8%)^37^. Among these, it was possible to identify different degrees of flocculation and also variable developmental behaviours. Out of 1973 strains, 29 flocculent ones were studied for their floc-forming ability using the differential effects of elevated temperature and proteases^38^. Subsequently, the genetic diversity of *FLO*1 and *FLO*5 in flocculent wine strains of *S. cerevisiae* was studied^29^. In addition, the same strains were characterised in terms of *FLO*1, *FLO*5, *FLO*8, *AMN*1 and *RGA*1 gene expression, growth kinetics and physicochemical properties of the cell surface during a 6-month sparkling wine fermentation period.

The aim of this work was to study the the importance/relevance of *FLO*1, *FLO*5 and *FLO*8 genes for flocculation in haploid strain F6789 that, as reported above, was found to possess higher flocculation ability (resistance to the action of four proteases) and high viability and resistance to sparkling wine fermentation stress. In addition, a strain that had lost its flocculation ability (F7101)^29, 38^, an autochthonous pulverulent strain isolated from Montepulciano d’Abruzzo wine (RT73)^39^ and strain 59A, a non-flocculent haploid derivative of Lalvin EC1118, were also used.

## Results

### Analysis of *FLO* genes distribution

**Figure 1:**
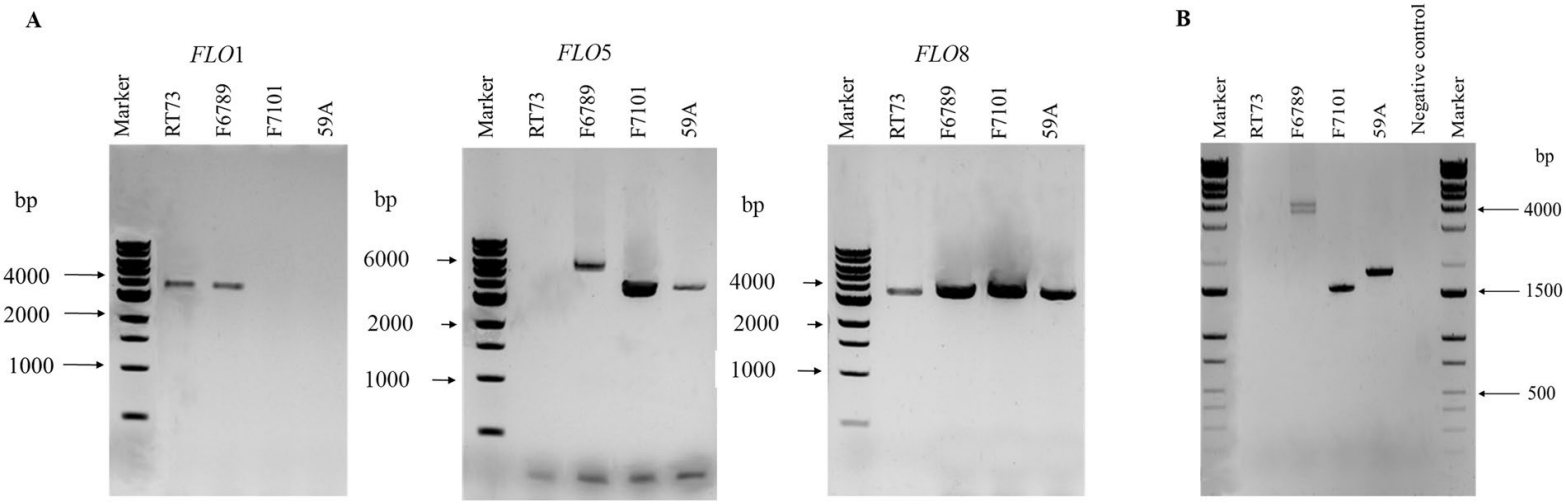
(**A**) Specific PCR detection of *FLO*1, *FLO*5 and *FLO*8 genes in *S. cerevisiae* strains. (**B**) Intragenic repetitive domains of gene *FLO*5. PCR mix without DNA was used as negative control.

The natural reservoir of *FLO* genes in *S. cerevisiae* strains was proposed as a reservoir to improve beverage industry because it allows green biomass separation^29, 40, 41^. For this reason, this study was first focused on *FLO*1, *FLO*5 and *FLO*8 genes in four *S. cerevisiae* wine strains (primers’ sequences and PCR conditions are reported in Supplementary Table S1). The whole genes were amplified using primers targeting zones before and after the coding regions. As shown in Fig. 1A, in all strains, the *FLO*8 gene displayed as expected a band with the same size of 3631 bp, confirming that this gene is present in all the *S. cerevisiae* strains studied. As regards the *FLO*1 gene, only two strains (pulverulent strain RT73 and flocculent strain F6789) had the same amplicon (approx. 3500 bp). No PCR products were observed for the pulverulent strains F7101 (yeast that has lost its flocculation ability) and 59A (industrial strain). The failure to amplify *FLO*1 gene in these two strains may be due to differences in sequences at the primer binding sites as also proposed by other authors or to the absence of the entire or part of the *FLO*1 gene sequence^28, 31^. In the same way, strain RT73 showed a short amplicon for gene *FLO*5, while the flocculent strain F6789 presented a 5000 bp amplicon, which was larger than in the other strains (about 4000 bp). All strains displayed a small band for *FLO*5 (approx. 150 bp), probably corresponding to a *FLO*-derived pseudogene. Many studies have demonstrated that the difference in length is due to the presence of tandem repeated sequences in the central region (B domain) of *FLO* genes^32^. Therefore, we investigated this domain of the *FLO*5 gene in our strains. As shown in Fig. 1B, three strains (59A, F6789 and F7101) exhibited at least one band for *FLO*5, while strain RT73 had no amplicon, because it either harbours a divergent gene in the primer sequence or just lacks gene *FLO*5. Interestingly, F6789 displayed two amplicons of approx. 4000 bp. Probably, the greater number of tandem repeats displayed by the flocculent strain F6789 hints to a central role of the *FLO*5 gene in the development of flocculation.

### *FLO* genes deletion and phenotype investigation

**Figure 2:**
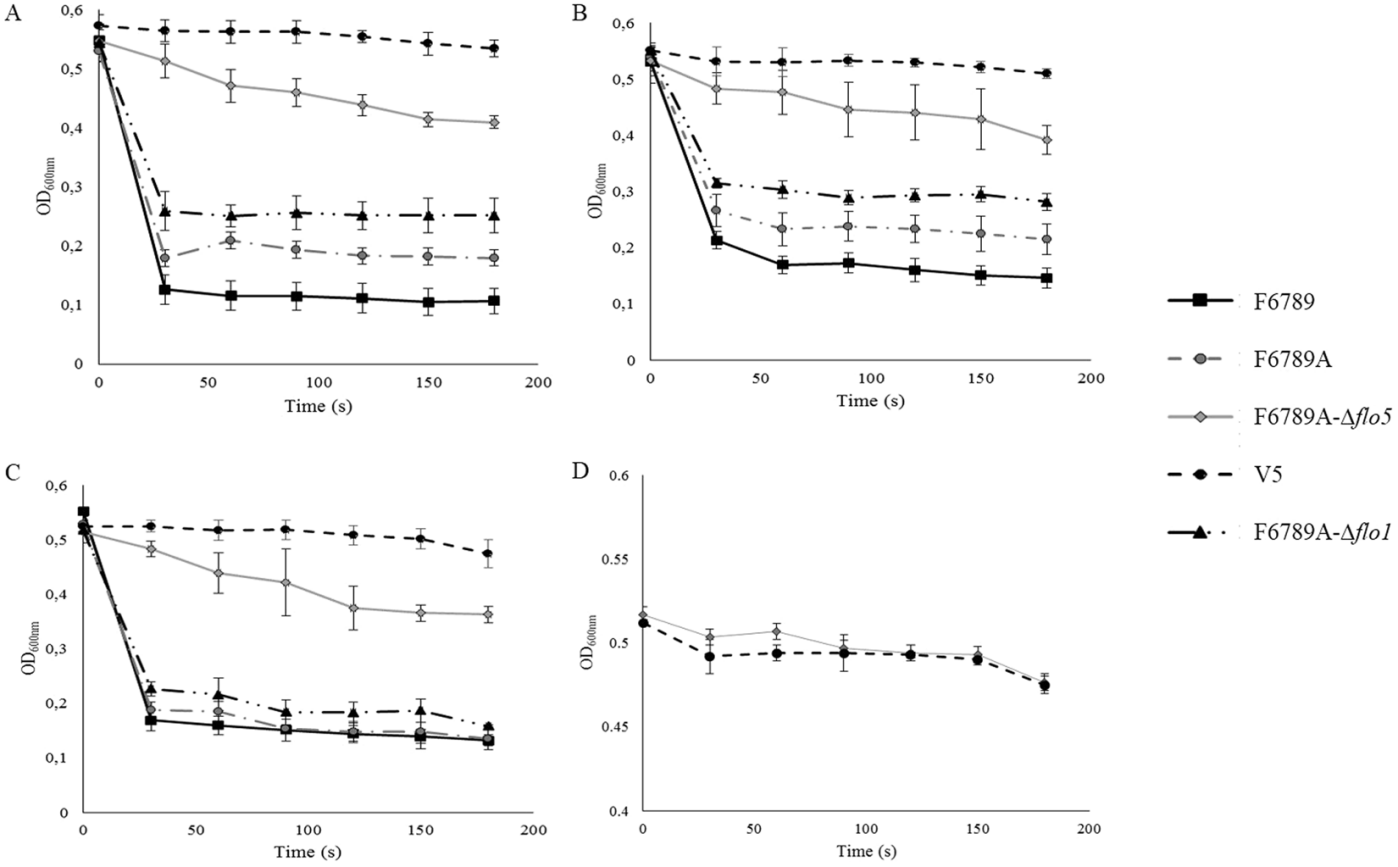
Flocculation level of parental (F6789), haploid (F6789A), transformants (F6789A*-*Δ*flo*1 F6789A*-*Δ*flo*5) and V5 strains. V5 is a laboratory haploid yeast derivative from a strain isolated from Champagne. This yeast was previously used as negative control for flocculation (Bidard *et al*.^45^) and we are sure that it has no adhesive properties. Flocculation was determined in YNB + glucose (2% w/v) (**A**), in YEPD (**B**) and in synthetic must (**C**). (**D**) Sedimentation without CaCl_2_ of non-flocculent strain V5 and F6789A-Δ*flo5*. The data reported are mean values for triplicates. Vertical error bars represent standard deviations.

To better understand which one of the *FLO* genes plays a major role in *S. cerevisiae* wine strains flocculation, we turned our attention to the F6789 strain. As this is a diploid strain, we obtained haploid clones by sporulation after deletion of the *HO* gene. This strain showed a sporulation ability of about 12%, with a 55% survival rate. As all the haploid clones obtained were flocculent, we selected the F6789A-Δ*ho* clone and both *FLO*1 and *FLO*5 genes were separately deleted. The resulting *FLO*-deleted haploid strains were subjected to a flocculation test in different media (YNB + glucose, YEPD and synthetic must). Results demonstrated that parental strain F6789 and its haploid derivative (F6789A) showed extensive flocculation whatever the medium used (Fig. 2A–C). In fact, these strains showed a decrease in OD_600__nm_ of about 0.4 in YNB + glucose, while the *FLO*1-deleted F6789A-Δ*flo*1 strain only displayed a weak reduction in OD_600__nm_ (≈0.329). By contrast, strains with a *FLO*5 deletion (F6789A-Δ*flo*5) exhibited a strong decrease in their flocculation ability and OD_600__nm_ dropped only by 0.14 in 3 min.

Interestingly, all flocculent strains and F6789A-Δ*flo*1 presented a hyperbolic curve with a strong OD_600 nm_ reduction already in the first 30 sec. Otherwise, F6789A-Δ*flo*5 strain showed a curve more similar to the non-flocculent strain (V5). In fact, these yeasts presented a slower and more gradual decrease in OD_600 nm_ than the parental, F6789A and F6789A-Δ*flo*1 strains. To investigate whether the residual aggregation was indeed flocculation, the same tests were conducted for strains F6789A-Δ*flo*5 and V5 without CaCl_2_ addition (Fig. 2D) as it is generally recognized that flocculation can only occur in the presence of Ca^2+^ ^17^. As expected, in this case, the strains had the same trend and the curves were superimposed, indicating that this is a typical Ca^2+^-dependent flocculation. Results demonstrated that in strain F6789, strong flocculation was strongly correlated with the expression of one specific *FLO* gene, *FLO*5.

**Figure 3:**
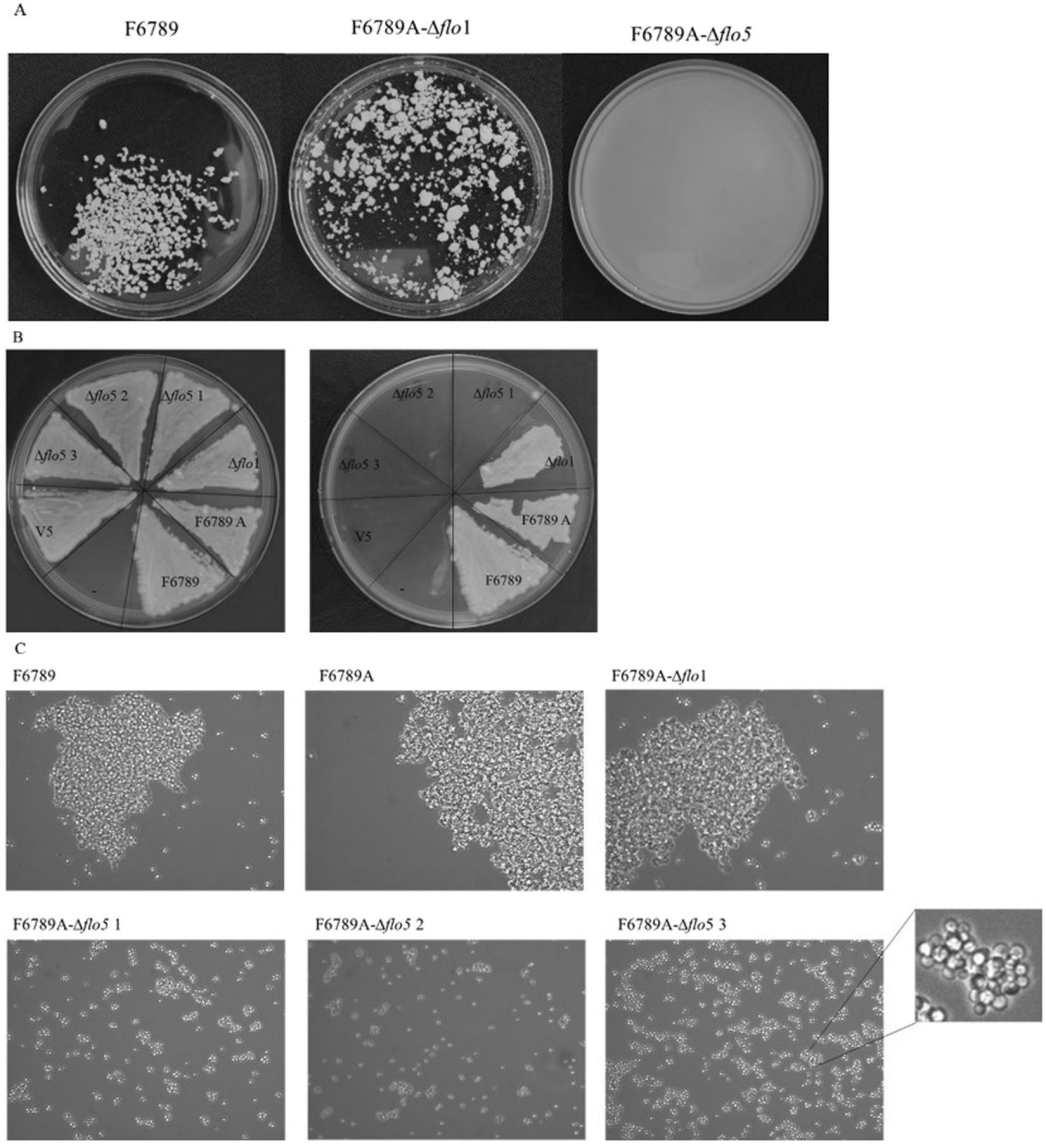
(**A**) Photographs of the parental (F6789), haploid (F6789A) and transformants F6789A-Δ*flo*1 and F6789A-Δ*flo*5 strains. Petri dishes were filled with equivalent volumes of YEPD medium originating from a liquid cell culture in stationary phase. (**B**) Agar invasion before and after the wash. Strains were spreaded onto YEPD plates and were cultivated for 3 days at 28 °C, followed by 2 days at room temperature. Plates were documented before and after non-adhesive cells were washed off the agar with a gentle stream of water. (**C**) Optical microscope photos (40X objective) of the parental strain (F6789), haploid strain (F6789A) and transformants.

To evaluate the residual flocculation of the transformants, cells were grown in YEPD for 48 h at 28 °C, after which flocs were observed with the naked eye and through an optical microscope (Fig. 3A and C). The parental strain presented, both at macroscopic and microscopic levels, large flocs of about 800000 μm^2^. The F6789A-Δ*flo*1 strain showed flocs slightly smaller than F6789 (about 650000 μm^2^). On the other hand, F6789A-Δ*flo*5 transformants were characterized by flocs unobservable to the naked eye. In fact, under the microscope, they presented flocs constituted of 20–30 cells with a 1600 μm^2^ size.

To evaluate the influence of *FLO*5 on other adhesion characteristics, an agar surface invasion analysis was conducted. Differences in the intensity of adhesion in F6789 derivatives were highlighted (Fig. 3B). Cells with *FLO*5 deletion were completely washed off the agar surface as fluffs after a short time of gentle washing. On the contrary, F6789A-Δ*flo*1 cells were not easily washed off the plate and a cell layer remained attached to the agar surface.

**Figure 4:**
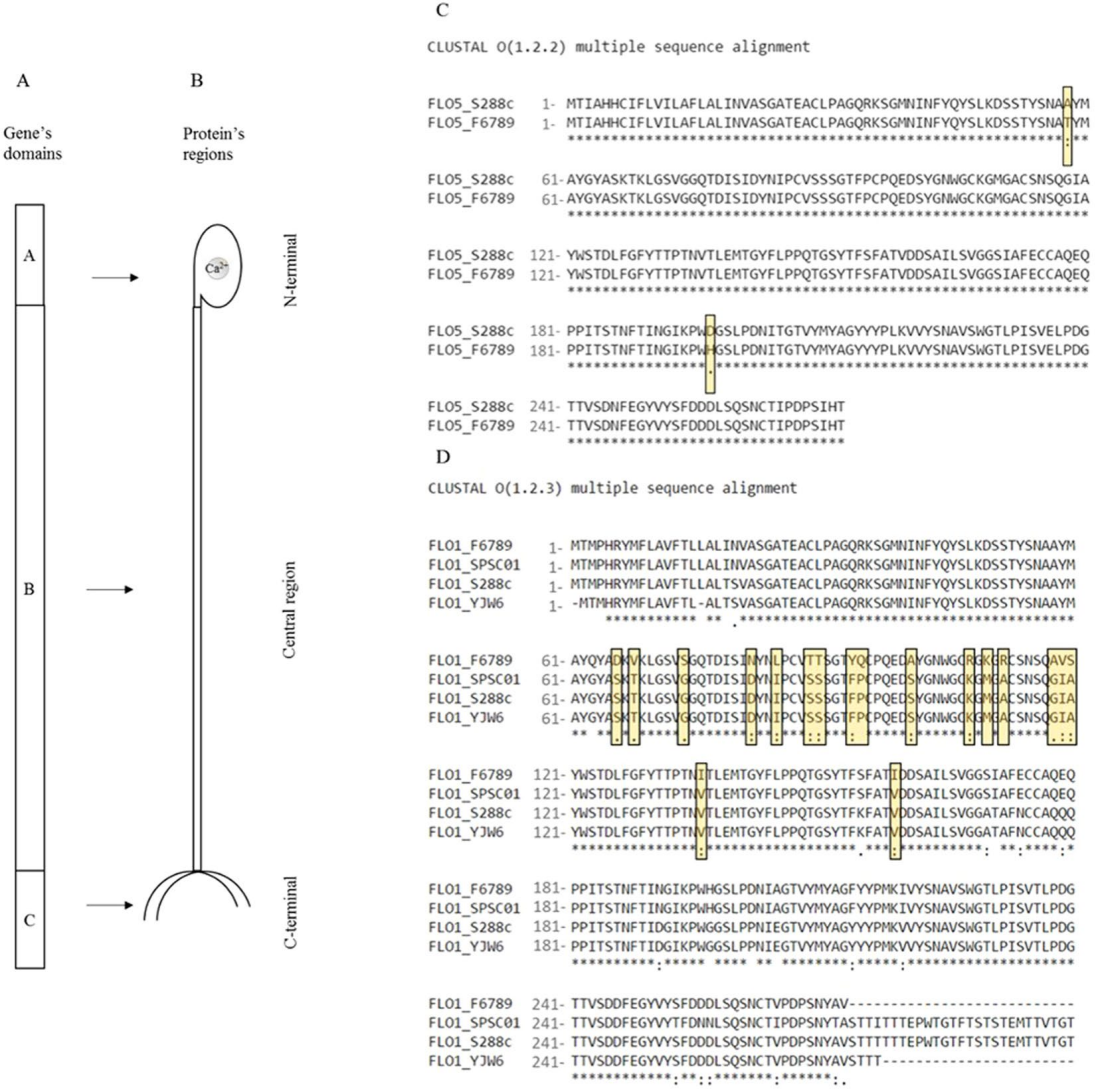
(**A**) Primary-structure of *FLO* genes. (**B**) Structure of Flo proteins. (**C**) Protein alignment of *FLO*5 protein between S288C and F6789A strains. Alignment with SPSC02 and YJW6 *FLO*5 sequences was not shown because these were not available in public databases. (**D**) Protein alignment of *FLO*1 protein between F6789A, SPSC01 (Accession n. JQ629938/protein id. AFJ20718.1)^38^, S288C and YJWE6 (Accession n.  X78160/protein id. CAA55024.1)^40^ strains.

To elucidate whether differences in the flocculation degree were due to a different binding strength of Flop, the N-terminal sequence of *FLO*1 and *FLO*5 was investigated. The DNA sequence of the *FLO*5 N-terminal gene was determined to be similar to the published S288C sequence except for two substitutions. The flocculent strain F6789 presented a guanine to adenine substitution, giving rise to a threonine instead of an alanine at position 58 (Fig. 4C). In position 197, the flocculent strain presented a histidine, where S288C has an aspartic acid residue, this difference being due to a guanine to cytosine substitution. Otherwise, the N-terminal sequence of *FLO*1 from *S. cerevisiae* F6789 showed only 83% similarity with the S288C N-terminal of Flo1p. Protein Flo1was aligned with the sequence of other two flocculent strains (Fig. 4D): the first one, SPSC01, is a protoplast fusant between *S. cerevisiae* and *Schizosaccharomyces pombe* used for fuel ethanol production^42^, while YJW6 is a haploid strain of *S. cerevisiae* with an inserted *FLO*1S gene obtained by Watari *et al*.^43^. F6789 Flo1p displayed 14 differences, showing 11 point mutations, 2 substitutions of 2 bases and 1 substitution of 3 bases.

### Segregation properties of flocculation

**Figure 5:**
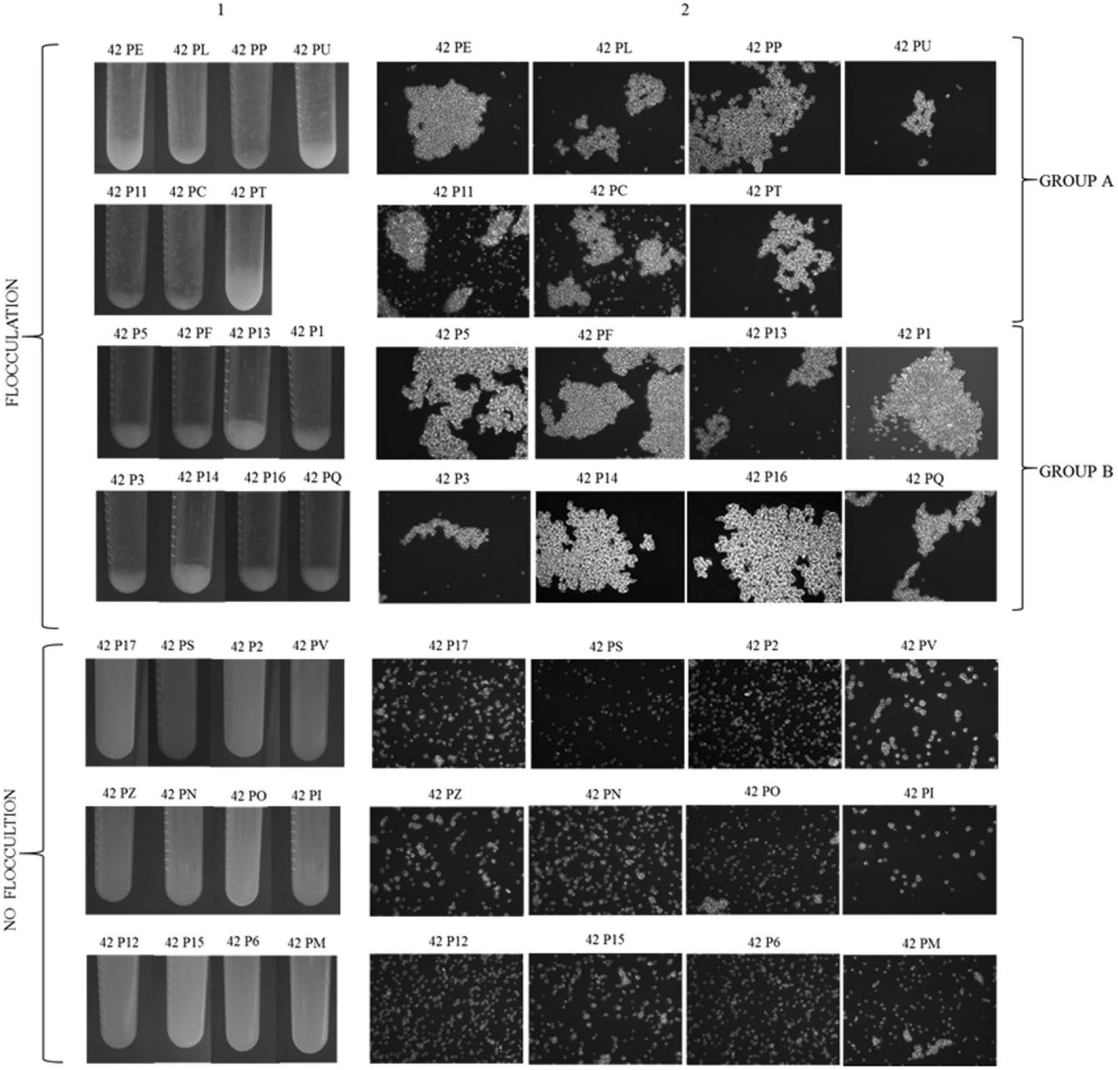
Phenotypic characterization of hybrids derived from the cross between 59A and F6789A strains. Column (**A**) sedimentation test: liquid cultures were vigorously mixed and placed in vertical position. Tubes were photographed after 30 s. Column (**B**) microscope observation (objective 40X).

In order to clarify the role of *FLO* genes in the F6789 strain, the segregation of the flocculation in a cross with a non-flocculent *S. cerevisiae* strain was monitored. Haploid strain F6789A-Δ*ho* was crossed with the non-flocculent *S. cerevisiae* strain 59A and the resulting hybrid (PDG 42) was induced to sporulate. After 5 days, PDG 42 presented 20% sporulation, with a 72% survival rate. Subsequently, the flocculation ability of segregants in YEPD medium was evaluated (Fig. 5). Sedimentation tests demonstrated that 55.6% of the strains were flocculent at different degrees. In particular, 2 different groups with low and high flocculation degrees were detected (Fig. 5 Column 1). Group A was composed of 7 segregants and group B included 8 strains that presented a high flocculation capacity. These results were confirmed by observations under an optical microscope (Fig. 5 Column 2). Cell size and number in flocs increased from the A to the B group. As described by Silva *et al*.^44^, all flocculent segregants presented opaque colonies with a rough aspect, irregular edges, and “castle” cross-section. On the contrary, about 44% of the isolates were pulverulent strains characterised by a dispersed development, a long sedimentation time and white, creamy colonies.

**Figure 6:**
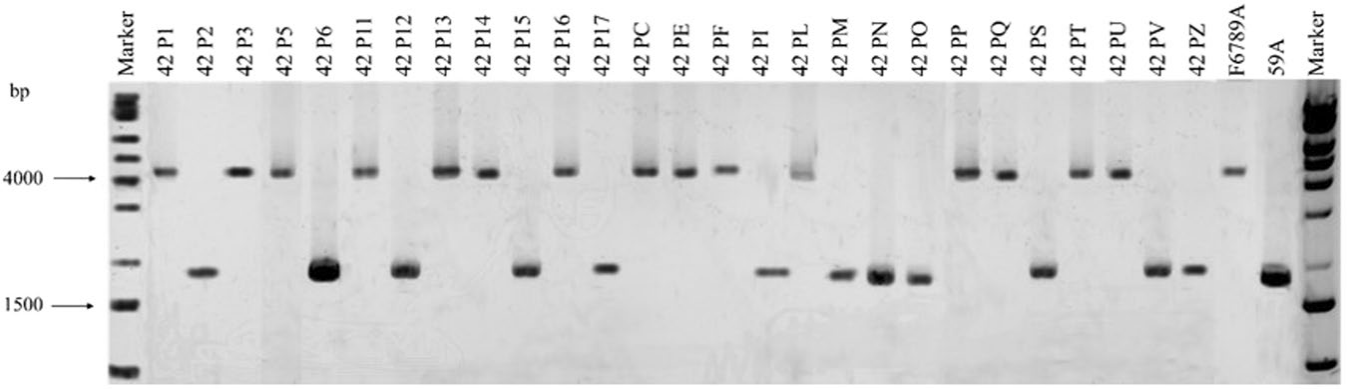
*FLO*5 gene repeated regions among segregants.

Then, *FLO*5-repeated regions in segregants were detected. Moreover, in order to have a more complete picture of *FLO* genes, *FLO*1 gene distribution was also investigated (Supplementary Table S2). Figure 6 clearly shows that all flocculation occurrences segregated with the *FLO*5 form of F6789A indicating that all flocculent strains had inherited their *FLO*5 gene from F6789A.

### Immunofluorescence

**Figure 7:**
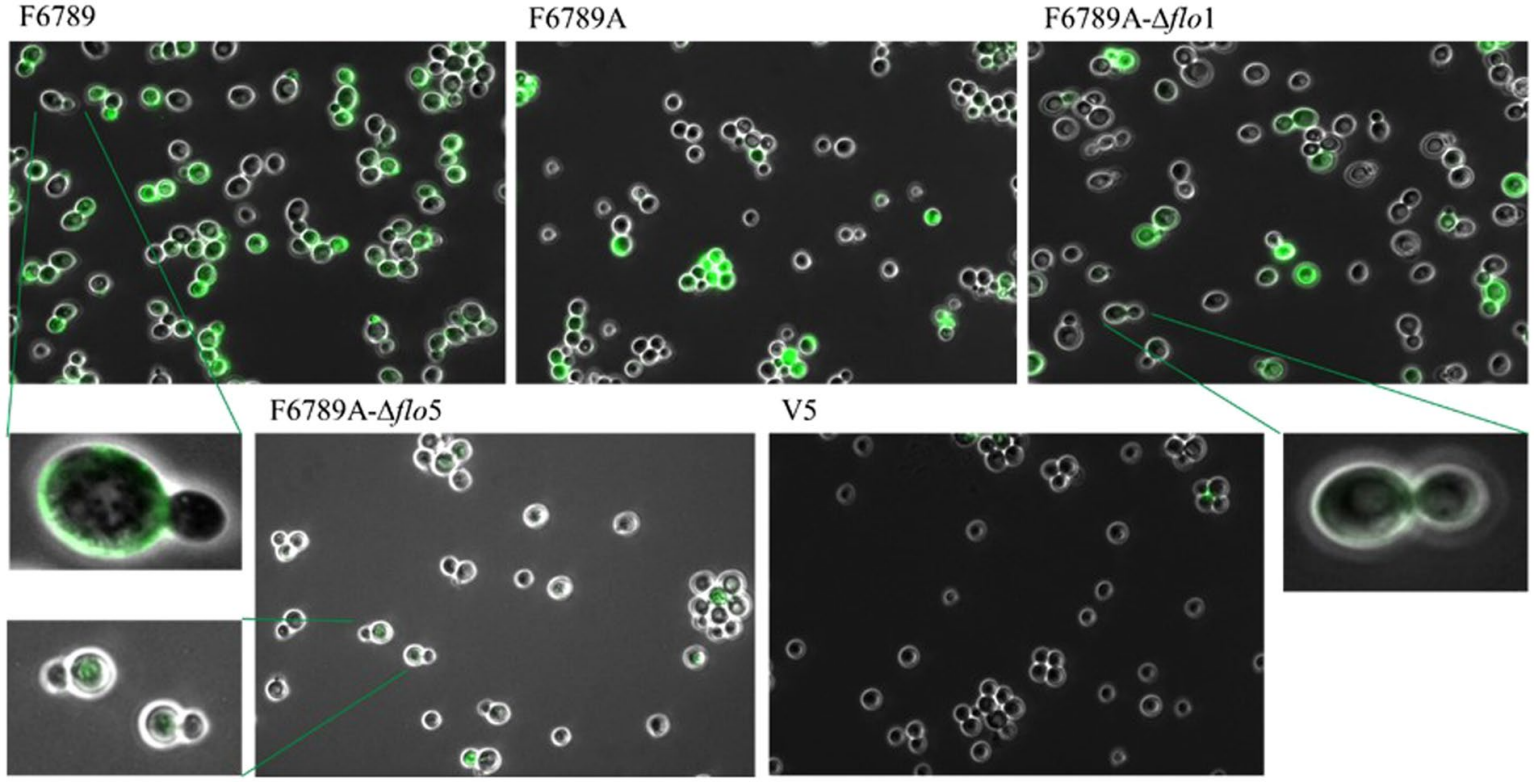
Localization of cell wall flocculins by FITC-labelled specific Anti-Flop antibody of parental (F6789), haploid (F6789A), F6789A-Δ*flo1*, F6789A-Δ*flo5* and V5 strains. The *S. cerevisiae* cells were grown in YNB+glucose. After 48 h (A_600 nm_ = 1), cells were stained with labelled lectin-specific IgGs and examined under a fluorescence microscope (100X oil objective). No fluorescence was observed in V5 strain nor in cells incubated with TBS only.

Flo proteins distribution at yeast cell surface was investigated by immunofluorescence staining with polyclonal antibodies. These antibodies, raised against Flo1p and obtained in a previous work^45^, are able to detect both proteins Flo1 and Flo5. Figure 7 shows that using FITC-labelled Anti-Flop, intact yeast cells of strains F6789 and F6789A could be stained during the stationary phase, thus indicating the presence of Flo proteins on their cell surface. However, flocculin distribution was not homogeneous: F6789 displayed approx. 70% stained cells, while only approx. 65% of the cells were stained in the F6789A strain. The strain harbouring a deleted *FLO*1 gene (F6789A-Δ*flo*1) displayed only a slight reduction in staining (about 10%). Consistently, the transformants harbouring a deletion of the *FLO*5 gene (F6789A-Δ*flo*5) presented an important reduction in staining with only 25% fluorescent cells. This strong reduction in Flo proteins detection with the anti-Flop antibodies is in line with a key role of *FLO*5 in triggering flocculation ability. It is interesting to note that in all studied strains there were cells that were not labelled and cells in which the reacting antigens were present only in specific points on the cell surface.

## Discussion

In *S. cerevisiae*, cellular aggregation depends on multiple genes controlled by diverse regulatory networks that determine the structure and composition of cellular surfaces^46^. Flocculation is strongly influenced by the expression of specific genes among which *FLO* genes play the main role^47^. In biotechnological applications, the possibility of inducing controlled flocculation in a naturally non-flocculent strain has great potential for improving brewing, winemaking, baking and ethanol-producing yeast strains^48^, so that studies on functional *FLO* genes are essential.

Initially, we focused our attention on three dominant flocculation genes, *FLO*1, *FLO*5 and *FLO*8^36^, in four *S. cerevisiae* wine strains, *i.e*. three non-flocculent strains (RT73, F7101 and 59A) and one flocculent strain (F6789). *FLO* family genes are present in all *S. cerevisiae* strains independently of their flocculation phenotype. Many studies, in fact, demonstrated that *FLO* genes are extraordinarily diverse in different laboratory, industrial and wine strains of *S. cerevisiae* ^29, 31, 32^. In this study, all wine strains presented genes of different lengths. *FLO*1 gene was similar in the pulverulent strain RT73 and in the flocculent strain F6789. As expected, the regulator gene *FLO*8 did not show different sizes in all studied strains. On the contrary, *FLO*5 size was particularly divergent among the tested strains, with repeated regions that ranged from 1500 bp to 4000 bp. Interestingly, *FLO*5-repeated regions in F6789 flocculent strain presented 2 bands, suggesting this gene is heterozygous in this diploid strain.

The variability in *FLO* genes length is due to two characteristics: position and structure. *FLO* genes (*FLO*1, *FLO*5, *FLO*9 and *FLO*10) are located in subtelomeric positions (∼10 to 40 kb from the telomeres)^20^ and contain repeated motifs. Genes residing near telomeres undergo frequent recombinations and duplications while, on the other hand, the central region of *FLO* genes contains several repeated motifs that provide a substrate for recombinations and generation of variation^49^. In the present study, all tested strains presented two amplicons for the whole *FLO*5 gene, one corresponding to the entire gene and the other likely to a pseudogene. Previously, strains F7101 and F6789 had just exhibited another pseudogene corresponding to the B domain of *FLO*1^29^. The presence of genes encoding homologues of one or several of the A, B or C domains is frequent in the *FLO* gene family^7^. Because these genes do not encode all three domains, they may have no function in cell surface adhesion^7, 49^, but they are important in terms of evolution. It was demonstrated for example that a recombination between the *S. cerevisiae*-type chromosome VIII and chromosome I of bottom-fermenting yeasts gave rise to Lg-*FLO*1^50^,the most important *FLO* gene in lager yeasts^49^.

We have focused our attention on which of the *FLO*1 and *FLO*5 genes in F6789 *S. cerevisiae* wine strain was responsible for the flocculation phenotype. Gene disruption experiments revealed that *FLO*5 is the most essential gene for flocculation since its deletion eliminated most of the ability to flocculate. *FLO*5-deleted strains were only able to form little flocs, which consisted in a few dozen cells slowly sinking down to the bottom of the tube. By contrast, deletion of *FLO*1 had no detectable impact on flocculation ability. A monitoring of flocculation ability after a cross with a non-flocculent strain revealed a clear co-segregation of the flocculation phenotype with the *FLO*5 gene inheritance. On the contrary, gene *FLO*1 from F6789A has proved incapable of ensuring flocculation but could increase flocculation degree in flocculating cells (Group B Figure 5, Supplementary Table S2). Therefore, flocculation in F6789 wine strain is controlled by *FLO*5, while *FLO*1 has only a marginal role in the determination of the degree of flocculation. These results suggest the possibly crucial role of Flo5p for cell-cell interactions in *S. cerevisiae* wine strain F6789.

The distribution of Flo proteins at the cell surface gave a picture consistent with a major role of Flo5p; *FLO*5 deletion induced a strong reduction in the number of fluorescent cells while *FLO*1 deletion only weakly affected immunofluorescence. These data strengthen the role of *FLO*5 driving flocculation in the F6789 strain. However, in strain F6789A-Δ*flo*5 only 25% of the cells exhibited some fluorescence. This finding suggests that the residual flocculation is the result of the expression of other flocculin(s), which could be driven at least in part by *FLO*1.

All considered strains showed non-homogeneous staining with completely unstained cells and cells coloured only in some parts of the cell wall. Our results are in agreement with other studies that demonstrated that flocculins are only expressed in a subpopulation within a community^6^. The authors highlighted that flocs can be formed by a mixed population of *FLO*1 and flo1 “cheater” cells that receive the protective benefits. Powell *et al*.^51^ suggested that the source of the observed difference in flocculation might be a variation in cell wall composition between young and old cells. They proposed that older cells, that are rougher than young cells and that express *FLO* genes, might act as nucleation points for floc formation. Other studies demonstrated that flocculins distribution on the cell surface is heterogeneous: El-Kirat-Chatel *et al*.^52^ evidenced that Flo1p is distributed in clusters at the cell surface. Furthermore, Bony *et al*.^53^ and Javadekar *et al*.^54^ observed an intense concentration of flocculins at the neck of the mother–daughter junction and of the buds.

In this study, we highlight the key role of the *FLO*5 gene in cell-surface adhesion. In fact, strains with a *FLO*5 deletion exhibited a non-adherent phenotype. It is generally recognized that biofilm formation, pseudohyphal and invasive growth depend of cell wall adhesin gene *FLO*11 (*MUC*1). Moreover, Torbensen *et al*.^55^ demonstrated that this gene could be replaced by another *FLO* gene. Furthermore, other studies showed that progressively deleting tandem repeats within *FLO*1 causes corresponding decreases in different adhesive phenotypes, such as adherence to plastic and flocculation^56^.

*S. cerevisiae* strain F6789 showed a high flocculation degree. This led us to investigate the nucleotide sequence of the A domain of Flo5p and Flo1p, i.e. the N-terminal region of the protein responsible for the binding with sugars. The N-terminal alignment of gene *FLO*5 between F6789 and S288C strains highlighted the presence of only two point mutations. As reported by Veelders *et al*.^17^, Flo proteins share high sequence identity; in particular the Flo5 A domain shares high similarity with the N-terminal sequence of Flo1 (94%), Flo9 (89%), and Flo10 (64%). Moreover, point mutations in the N-terminal of Flo5p can increase or decrease substrate affinity determining different binding strengths^17^. Otherwise, when we compared F6789 Flo1p sequence with other flocculent and laboratory strains, several mutations in the N-terminal region were found. Haploid segregants obtained with the flocculent hybrid PDG 42 that had inherited the *FLO*1 gene from F6789 were not able to achieve flocculation. It could be due to the mutations in this region or to the different expression of Flo1p^29, 40^. Further studies are necessary to explain these data.

Except for the *FLO* genes in model yeast strains such as S288C, our current knowledge of the natural diversity of *FLO* genes is still very limited^28^. Even if *FLO*1 and *FLO*5 are paralogues^57, 58^ and even if the molecular basis of flocculation is essentially identical, Watari *et al*.^59^ speculated that the flocculation properties of most industrial flocculent strains might derive from *FLO*5 gene expression. Indeed, all the industrial flocculent strains that they examined were heat-sensitive and chymotrypsin-resistant, indicating that the flocculence phenotype could be determined by the *FLO*5 gene.

Our study confirms this hypothesis; in fact, we found that in flocculent *S. cerevisiae* wine strain F6789, the *FLO*5 gene drives flocculation phenotype. In fact, a *FLO*5 deletion causes a strong reduction in flocculation ability. Furthermore, this study shows that *FLO*5 influences the adhesive properties of this flocculent yeast. This specific case confirms that the role of a given flocculin can also be played by another flocculin because the members of this family have partial functional redundancy^60^. Random events, related to yeast evolution in different environments or in response to specific interspecies association within the microbial ecosystem^33^, most probably led to the expansion of a specific *FLO* gene that would become essential for flocculation in a given strain.

## Materials and Methods

### *Saccharomyces cerevisiae* strains

In this study 4 *S. cerevisiae* strains were used: a flocculent strain (F6789), a strain that had lost this capacity (F7101)^29, 38^, a pulverulent strain isolated from Montepulciano d’Abruzzo wine^39^, and a non-flocculent strain (59A, a haploid derivative of Lalvin EC1118). The wine strain V5 was used as negative control. Deletion mutants were originated from F6789 (reference parental strain).

To obtain a haploid strain from F6789, the *HO* gene was deleted using the lithium acetate method^61^ (sequences primers *HO* del F and *HO* del R^62^ reported in Supplementary Table S1). The G418 resistance marker kanMx4 from the pUG6 plasmid was used. Spores were isolated by micromanipulation (Singer Instruments, Roadwater, United Kingdom). Stable haploid spores disrupted for the *HO* gene were selected on YEPD plates supplemented with the corresponding antibiotic (G418 at 200 μg/mL*)*.

The mating type of the haploids was determined through crossing experiments with reference strains of known mating type. The F6789A-Δ*ho (*MATα) strain was selected for further analysis.

### *FLO* genes deletion

The flocculent *S. cerevisiae* haploid strain F6789A-Δ*ho* was transformed with two different plasmids as reported above. The *FLO*1-deleted transformant, F6789A-Δ*flo1*, was obtained using a PCR-amplified fragment of the pAG25 plasmid (NatMx4 cassette) carrying the selective marker for nourseothricin, and primers *FLO*1 del F and *FLO*1-5 del R (Supplementary Table S1). For *FLO*5 deletion (F6789A-Δ*flo5*) the pAG32 plasmid (containing the hygromycin resistance) was used and amplification was performed with *FLO*5 del F and *FLO*1-5 del R (Supplementary Table S1). The reaction was performed in a total volume of 50 μL using the Ex Taq™ kit (Ex Taq™, TAKARA BIO INC., Otsu, Shiga, Japan). Hygromycin or nourseothricin-resistant colonies were analysed by PCR to confirm the correct genomic integration of the cassette.

### Media and culture conditions

Yeast pre-cultures were done by selecting a colony from cultures on fresh yeast extract-peptone-dextrose (YEPD) agar plates (10 g/L Bacto-yeast extract, 20 g/L Bacto-peptone and 20 g/L dextrose). Sporulation was induced on spoMA medium (1 g/L Bacto-yeast extract, 0.5 g/L glucose, 10 g/L potassium acetate and 20 mg/L adenine) at 28 °C. Transformed strains were selected and grown on YEPD medium supplemented with 200 mg/L geneticin (Sigma-Aldrich, Lezennes, France), hygromycin (Sigma-Aldrich) or nourseothricin (Sigma-Aldrich)^63^. Flocculation degree was quantified in YNB+glucose (YNB 0.67% w/v, glucose 2% w/v), YEPD and synthetic must MS425^64^. Cells were stored in YEPD broth supplemented with glycerol (Sigma-Aldrich, 20% v/v final concentration) at −80 °C.

### PCR amplification of *FLO*1, *FLO*5 and *FLO*8 genes

Primers used in this study are listed in Supplementary Table S1. Based on the S288C genome, primers were designed using the Unipro UGENE software 1.19.0 to selectively amplify the entire sequence of *FLO*1, *FLO*5 and *FLO*8 genes. To develop oligos for *FLO*1, *FLO*5 and *FLO*11, repeated regions in the long intragenic repeats (>40 nt) were identified using the EMBOSS ETANDEM software26 with the threshold score set at 20. Primer specificity was checked with a BLAST analysis of all *FLO* genes from strain S288C.

Total genomic DNA was extracted using the Wizard^®^ Genomic DNA Purification Kit (Promega Corporation, Charbonnières-les-Bains, Lyon, France), according to the manufacturer’s instructions. PCR amplification reactions were performed in a total volume of 25 μL. Each reaction mixture contained 20 ng template DNA, 10 X Ex Taq™ buffer (Ex Taq™, TAKARA BIO INC.) containing 4.5 mM MgSO_4_, 4 μL of 10 mM dNTPs, 20 pmol each of the amplification primers and 0.20 μL Takara ex Taq DNA Polymerase. PCR was performed using a TRIO Thermal Cycler (Biometra, Göttingen, Germany). PCR products were visualised on a 1% agarose gel and acquired using the Kodak GL 100 imaging system (Fisher Scientific, Illkirch-Graffenstaden, France). Gel conversion, normalisation and analysis were carried out using Fingerprinting II Informatix™ Software (Bio-Rad, Milan, Italy). PCR products were quantified with a NanoDrop 1000 (Thermo Fisher Scientific, Villebon-sur-Yvette, France), and purified by NucleoSpin Gel and PCR clean-up kit (Macherey Nagel SARL, Sète, France) for sequence analysis.

The N-terminal sequence of *FLO*1 (primers *FLO*1 A, *FLO*1 BF and *FLO*1 BR) and *FLO*5 (primers *FLO*5-AF and *FLO*5-AR, *FLO*5-BF and *FLO*5-BR) genes was amplified using the KAPA HiFi PCR Kits (Kapa Biosystems, Nanterre, France), according to the manufacturer’s instructions. PCR products were sequenced (Eurofins MWG GmbH, Martinsried, Germany) and the resulting sequences translated using the «Expasy Translate Tool» (http://www.expasy.org/tools/dna.html) program. Alignments were made using program Clustal omega (http://www.ebi.ac.uk/Tools/msa/clustalo).

### Ploidy level of *S. cerevisiae* strains

Strain ploidy level was assessed using SYTOX® Green (Invitrogen) fluorescence and flow cytometry according to Delobel and Tesnière^65^, using an Accuri C6^TM^ flow cytometer. A single colony was selected and grown overnight in liquid YEPD with shaking at 150 rpm. Cells in exponential phase were collected by centrifugation and then re-suspended in 1 mL water. Eight ml of 75% ethanol were added and fixation was performed overnight at 4 °C. Fixed cells were harvested by centrifugation and washed with 1 mL PBS 1X. Cells were re-suspended in 500 μL RNAse A (Qiagen, Paris, France) solution (2 mg/mL RNAse A in 10 mM Tris-HCl, 15 mM NaCl) and incubated at 37 °C for 1 h and finally re-suspended in 200 μL of proteinase K (Roche, Paris, France) solution (1 mg/Ml in PBS 1X). After a 1 h- incubation at 50 °C, cells were collected by centrifugation, re-suspended in 500 μL PBS 1X, sonicated for 15 s, and stored on ice until analysis. Fifty µL of cell suspension were added to 200 μl of SYTOX® Green solution (1.25 µM SYTOX® Green in PBS 1X). Analysis was performed with a Multisizer 3 Particle Counter (Beckman Coulter, Paris, France). The FL1 detector (530/30 nm) was used for the acquisition of SYTOX® Green fluorescence, as this dye is taken up by cells in a manner stoichiometric to the amount of nuclear DNA.

### Phenotype investigation

#### Flocculation trials

Flocculation was quantified by measuring the optical density of yeast suspensions after shaking^45^. Yeast cells were harvested after 48 h, washed twice in deflocculation buffer (5 mM EDTA; 50 mM sodium citrate pH = 3) and suspended in the same buffer at the final concentration of 5 × 10^7^ cells/mL. Flocculation was induced by addition of CaCl_2_ (20 mM) to 5 mL of the cell suspension. The tubes were placed on a shaker and agitated at 50 oscillations/min, for 5 min at room temperature. An aliquot of 200 μL of the upper phase was withdrawn and 1 mL of 250 mM EDTA (pH = 8) was added. The optical density at 600 nm was measured immediately and at 30 s intervals for 3 min using a spectrophotometer (Spectronic Model 601, Spectronic Instruments, Rochester, NY, USA). Three individual transformants were used to generate the data with regard to the expression of *FLO*5 truncations, while only one transformant for *FLO*1 deletion. All analyses were performed in triplicate.

### Invasive growth assay

Invasive growth assay was performed as previously described by Tofalo *et al*.^29^. A fresh culture of each strain was isolated on YEPD plates with a toothpick and incubated for 3 days at 28 °C and then for 2 days at room temperature. Deionized water was used to wash all cells off the agar surface, leaving subsurface cells that had actually invaded the agar. Plates were photographed before and after washing. All analyses were performed in triplicate.

### Microscopy

Cells were examined using an Axio Imager.A2 epifluorescent microscope (Zeiss, Carl Zeiss Inc., Thornwood, NY, USA).

### Cross

Diploid strains were originated by mating 59A (MATa) and F6789A-Δ*ho* (MATα) haploid cells and the selection of zygotes was done by micromanipulation (Singer Instruments). Zygote ploidy was tested by flow cytometry as reported above. A diploid hybrid was sporulated and tetrads containing the four haploid meiotic products were dissected. Twenty-seven segregants were considered for further analyses.

### Phenotype and genotype of cell aggregation

To test flocculation, cells were cultured in YEPD liquid medium at 28 °C at 240 rpm for 48 h to make the cells enter the stationary phase of growth. Cultures were swirled briefly for 30 s with a vortex shaker before the sediment test. Cells were photographed after 30 s in vertical position. *FLO*5 repeated regions were investigated as reported above.

### Immunofluorescent staining

Immunofluorescent staining was carried out according to Bidard *et al*.^45^. The antiserum was pre-adsorbed with V5 strain and was used at a 1:10 dilution. After deflocculation (0.5 M EDTA) stationary-phase yeast cells (10^7^ cells/mL) were centrifugated and suspended in TBS buffer (10 mM Tris-HCl, 140 mM NaCl, 5 mM EDTA, 20 μg/mL cycloheximide). Thirty μL of the antiserum were added and shakenfor 75 min. Cells were harvested by centrifugation and washed three times in TBS buffer. Thirty μL fluorescein isothiocyanate (FITC)-conjugated goat anti-rabbit IgG (Sigma–Aldrich) diluted (1:160) in the same buffer were added. After 30 min shaking, cells were washed three times in the same buffer and 10 μL water containing 1 mg/mL of p-phenylenediamine were added. Cells were photographed by immunofluorescence microscopy (Zeiss Axio Imager.A2). The light was set at 521 nm and a 100X oil objective was used.

## Electronic supplementary material

Supplementary Table S2

Supplementary Table S1
