# Supplementary material for: FLO5 gene controls flocculation phenotype and adhesive properties in a Saccharomyces cerevisiae sparkling wine strain
Source: Sci Rep. 2017 Sep 7;7:10786. doi: 10.1038/s41598-017-09990-9 (PMC5589750; doi:10.1038/s41598-017-09990-9)
Supplement: Supplementary file 1 — Supplementary Table S2 [file 41598_2017_9990_MOESM1_ESM.doc]

***FLO*5 gene controls flocculation phenotype and adhesive properties in a *Saccharomyces cerevisiae* sparkling wine strain**

**Paola Di Gianvito^1^, Catherine Tesnière^2,3,4^, Giovanna Suzzi^1*^, Bruno Blondin^2,3,4*^, Rosanna Tofalo^1^**

^1^ Faculty of BioScience and Technology for Food, Agriculture and Environment, University of Teramo, Via R. Balzarini 1, 64100 Teramo, Italy

^2^ INRA, UMR1083 Science pour l’Œnologie, Montpellier, France;

^3^ Montpellier SupAgro, UMR1083 Science Pour l’Œnologie, Montpellier, France;

^4^ Université Montpellier 1, UMR1083 Science pour l’Œnologie, Montpellier, France

**Supplementary Table S2**. Inheritance of *FLO*5 and *FLO*1 genes in the segregants investigated in the manuscript. A three-level scale was used for flocculation degree evaluation: 0 non-flocculent strain, 1 presence of little flocs, 2 big flocs formation

| Strain | *FLO*5 | *FLO*1 | Flocculation degree |
| --- | --- | --- | --- |
| 42 P17 | 59A | 59A | 0 |
| 42 PS | 59A | 59A | 0 |
| 42 P2 | 59A | 59A | 0 |
| 42 PV | 59A | 59A | 0 |
| 42 PZ | 59A | 59A | 0 |
| 42 PN | 59A | 59A | 0 |
| 42 PO | 59A | 59A | 0 |
| 42 P6 | 59A | F6789 | 0 |
| 42 PI | 59A | F6789 | 0 |
| 42 P12 | 59A | F6789 | 0 |
| 42 P15 | 59A | F6789 | 0 |
| 42 PM | 59A | F6789 | 0 |
| 42 PL | F6789 | 59A | 1 |
| 42 PP | F6789 | 59A | 1 |
| 42 PU | F6789 | 59A | 1 |
| 42 PE | F6789 | 59A | 1 |
| 42 PC | F6789 | 59A | 1 |
| 42 PT | F6789 | 59A | 1 |
| 42 P11 | F6789 | 59A | 1 |
| 42 P5 | F6789 | F6789 | 2 |
| 42 PF | F6789 | F6789 | 2 |
| 42 P13 | F6789 | F6789 | 2 |
| 42 P1 | F6789 | F6789 | 2 |
| 42 P3 | F6789 | F6789 | 2 |
| 42 P14 | F6789 | F6789 | 2 |
| 42 P16 | F6789 | F6789 | 2 |
| 42 PQ | F6789 | F6789 | 2 |
