# Supplementary material for: FLO5 gene controls flocculation phenotype and adhesive properties in a Saccharomyces cerevisiae sparkling wine strain
Source: Sci Rep. 2017 Sep 7;7:10786. doi: 10.1038/s41598-017-09990-9 (PMC5589750; doi:10.1038/s41598-017-09990-9)
Supplement: Supplementary file 2 — Supplementary Table S1 [file 41598_2017_9990_MOESM2_ESM.doc]

***FLO*5 gene controls flocculation phenotype and adhesive properties in a *Saccharomyces cerevisiae* sparkling wine strain**

**Paola Di Gianvito^1^, Catherine Tesnière^2,3,4^, Giovanna Suzzi^1*^, Bruno Blondin^2,3,4*^, Rosanna Tofalo^1^**

^1^ Faculty of BioScience and Technology for Food, Agriculture and Environment, University of Teramo, Via R. Balzarini 1, 64100 Teramo, Italy

^2^ INRA, UMR1083 Science pour l’Œnologie, Montpellier, France;

^3^ Montpellier SupAgro, UMR1083 Science Pour l’Œnologie, Montpellier, France;

^4^ Université Montpellier 1, UMR1083 Science pour l’Œnologie, Montpellier, France

**Supplementary Table S1.** Primers used in this study

| **Primer** | **Sequence 5’-3’** | **Comments** | **Annealing temperature (°C)** | **Sources** |
| --- | --- | --- | --- | --- |
| *FLO*1 CF | TGACAATGCCTCATCGCTAT | On the promoter of *FLO*1 gene | 57 | This study |
| *FLO*1 A | GAAAGGCACTCAAAAAGATAAATCA | upstream *FLO*1 gene |  | Euroscarff primer list |
| *FLO*1 D | AATAGGGCATTTTTCCTCGTTAATA | downstream *FLO*1 gene | 57 | Euroscarff primer list |
| *FLO*t5 F | GAGGCACTGTTCCTTCCCAA | upstream *FLO*5 gene | 60 | This study |
| *FLO*t5 R | CACAGATACGTAAAAAGAACGCGA | downstream *FLO*5 gene | 60 | This study |
| *FLO*8 A | CAACGAGTGTATAGTGCATGAAATC | upstream *FLO8* gene | 60 | Euroscarff primer list |
| *FLO*8t R | GCTTTCATGCTGCGCTCTAC | downstream *FLO*8 gene | 60 | This study |
| *FLO*1r-F | GACTTCGAAGGGTACGTC | Repeated region *FLO*1 gene | 57 | 42 |
| *FLO*1r-R | GAATGGGGTAATAATTGGACG | Repeated region *FLO*1 gene | 57 | 42 |
| *FLO*5r-F | GTTTACTCTTTTGACGATGACCT | Repeated region *FLO*5 gene | 58 | This study |
| *FLO*5r-R | GTTGTCTCACTGGTAGCACTG | Repeated region *FLO*5 gene | 58 | This study |
| *FLO*11-Reps F | TAGTGCCGCTCAATATGCAAGCTCCTGGCA | Repeated region *FLO*11 gene | 65 | 32 |
| *FLO*11-Reps R | TGTTTGACTGCCAGGGTATTTGGATGATG | Repeated region *FLO*11 gene | 65 | 32 |
| *HO* del FOR | ATGCTTTCTGAAAACACGACTATTCTGATGGCTAACGGTG CTTCGTACGCTGCAGGTC | upstream *HO* gene | 60 | 43 |
| *HO* del REV | TTAGCAGATG CGCGCACCTG CGTTGTTACC ACAACTCTT TAGTGGATCT GATATCACCT A | downstream *HO* gene | 60 | 43 |
| *FLO*1 BF | TTCACCATCAATGGTATCAAGC | N- terminal *FLO*1 gene | 55 | This study |
| *FLO*1 BR | GAATAGACGTACCCTTCGAAGTCA | N- terminal *FLO*1 gene | 55 | This study |
| *FLO*5 AF | TACGCGAAATGCAGCATAAG | N- terminal *FLO*5 gene | 60 | This study |
| *FLO*5 AR | AACAAGCACCCATTCCTTTG | N- terminal *FLO*5 gene | 60 | This study |
| *FLO*5 BF | TCCTCCACATATTCGAAT | N- terminal *FLO*5 gene | 60 | This study |
| *FLO*5 BR | GTAGTTGTGATAGTGCTA | N- terminal *FLO*5 gene | 60 | This study |
| *FLO*1del F | ATGACAATGCCTCATCGCTATATGTTTTTGGCAGTCTTTAttcgtacgctgcaggtcgac | Deletion *FLO*1 gene | 60 | This study |
| *FLO*1-5 del R | TTAAATAATTGCCAGCAATAAGGACGCAATGAAGACACACTgcataggccactagtggatctg | Deletion *FLO*1- *FLO5* gene | 60 | This study |
| *FLO*5 del F | ATGACAATTGCACACCACTGCATATTTTTGGTAATCTTGGTTCGTACGCTGCAGGTCGAC | Deletion *FLO5* gene | 60 | This study |
